# Supplementary material for: Role of the osaA Gene in Aspergillus fumigatus Development, Secondary Metabolism and Virulence
Source: J Fungi (Basel). 2024 Jan 26;10(2):103. doi: 10.3390/jof10020103 (PMC10890407; doi:10.3390/jof10020103)
Supplement: Supplementary file 1 [file jof-10-00103-s001.zip › jof-2810382-supplementary.pdf]

Role of the *osaA* gene in *Aspergillus fumigatus* development, secondary metabolism and virulence

Apoorva Dabholkar[1], Sandesh Pandit[1], Ritu Devkota[2], Sourabh Dhingra[2], Sophie Lorber[3], Olivier Puel[3], Ana M. Calvo[1*]

[1] Department of Biological Sciences, Northern Illinois University, DeKalb, Illinois, United States of America

[2] Department of Biological Sciences and Eukaryotic Pathogen Innovation Center, Clemson University, Clemson, South Carolina, USA

[3] Toxalim (Research Centre in Food Toxicology), Université de Toulouse, INRAE, ENVT, INP-Purpan, UPS, Toulouse, France

*Corresponding author

Email: amcalvo@niu.edu

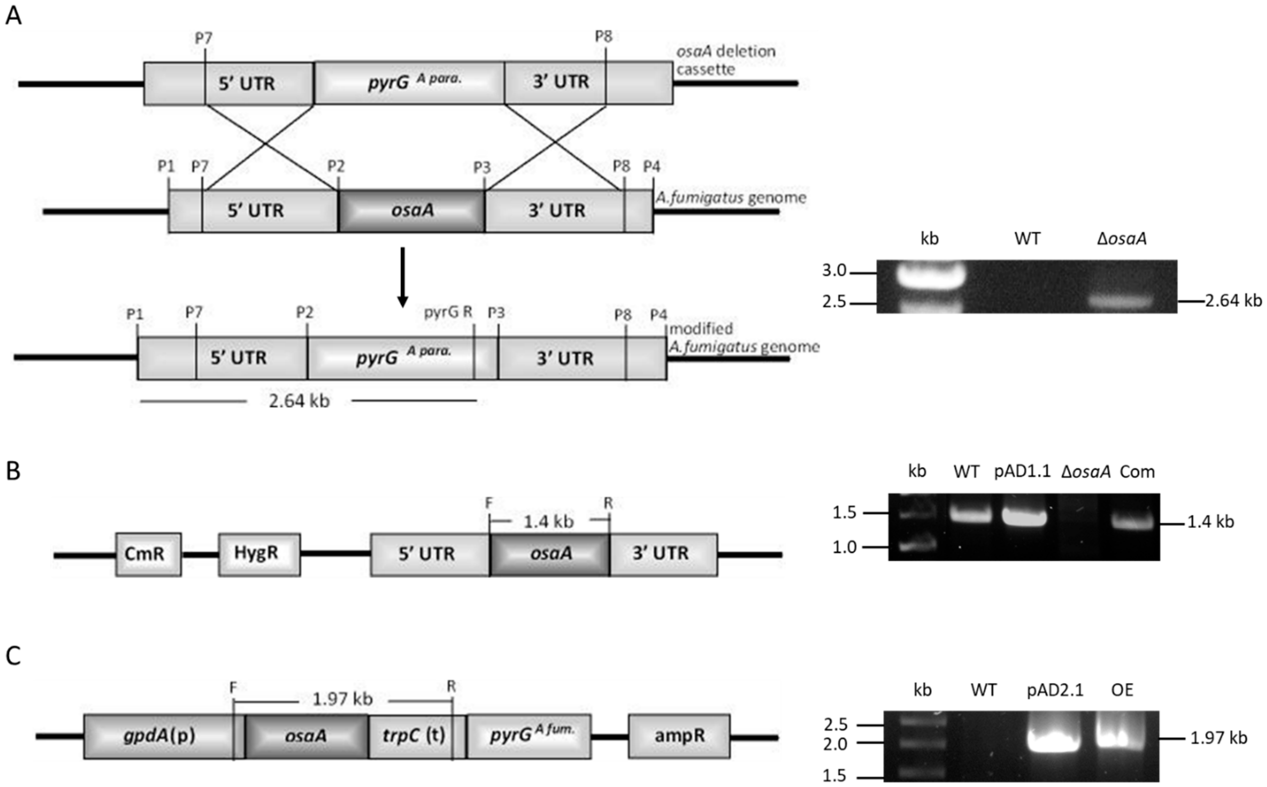

Figure S1. Generation of the *osaA* deletion, complementation and overexpression strains. (A) Generation of the *osaA* deletion strain (Δ*osaA*). Diagram showing the replacement of *osaA* gene with the *A. parasiticus pyrG* marker gene by homologous recombination. On the right, diagnostic PCR using primers P1 (osaA_F1) and *pyrG*R (A para pyrG_R) resulting in the expected 2.64 kb PCR product. Wild-type strain was used as negative control. (B) Generation of the *osaA* complementation strain (Com). Schematic representation of *osaA* complementation plasmid (pAD1.1). The hygromycin resistance gene, *hygR*, was used as a transformation marker. On the right, confirmation of the complementation transformant by diagnostic PCR using primers *osaA*F (osaA_OE1) and *osaA*R (osaA_OE2). The expected PCR product of 1.4 kb was observed. Wild type and pAD1.1 were used as positive controls, *osaA* deletion strain was used as negative control. (C) Generation of the *osaA* overexpression strain (OE). Representation of the *osaA* complementation plasmid (pAD2.1). On the right, diagnostic PCR confirmation of *osaA* OE strain, showing the expected 1.97 kb product amplified with primers *gpdA*F (gpdA_F) to *trpC* (trpC_R). pAD2.1 was used as positive control, and wild type was used as negative control.

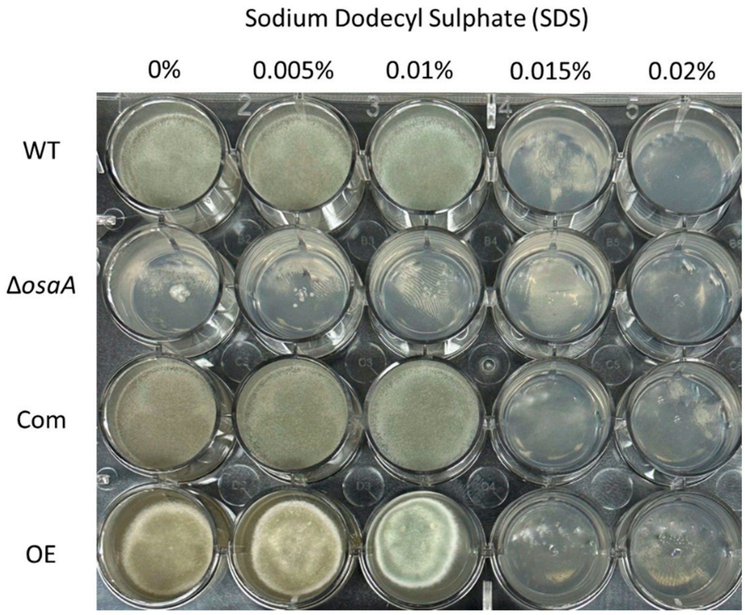

Figure S2. Deletion *osaA* is more susceptible than wild type to the presence of sodium dodecyl sulphate (SDS). *A. fumigatus* strains were point-inoculated on GMM and GMM supplemented with a range of sodium SDS concentrations. The plates were incubated at 37°C for 5 days. The experiment was performed in triplicates.

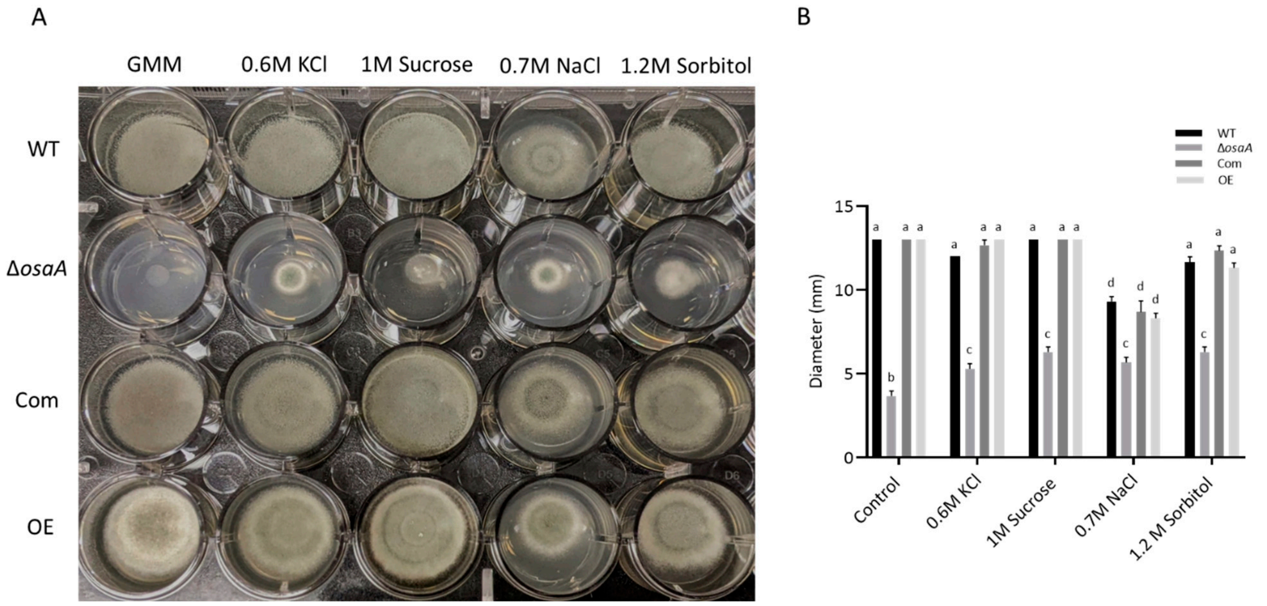

Figure S3. Growth reduction of the *A. fumigatus* Δ*osaA* strain is partially remediated in the presence of osmotic stabilizers. (A) *A. fumigatus* wild type (WT), deletion *osaA* (Δ*osaA*), complementation (Com) and overexpression (OE) strains were point-inoculated on GMM and GMM supplemented with 0.6 M KCl, 1 M sucrose, 0.7 M NaCl or 1.2 M sorbitol. Plates were incubated at 37°C for 72 h. The experiment was carried out in triplicate. (B) Colony diameter measurement of *A. fumigatus* cultures. Error bars indicate the standard error. Different letters on the columns indicate values that are statistically different ($p < 0.05$). All the experiments were done in triplicates.

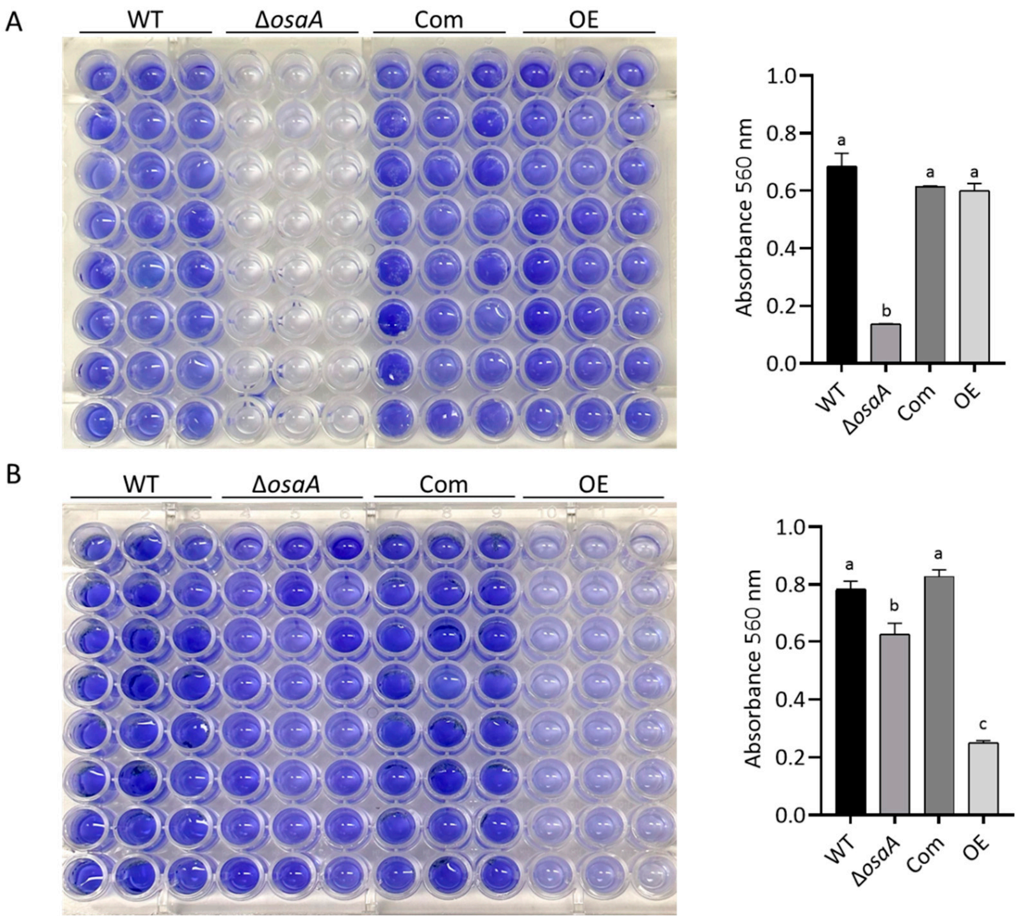

Figure S4. *osaA* is necessary for normal adhesion to solid surfaces in *A. fumigatus*. Wild type (WT), Δ*osaA,* complementation (Com) and overexpression (OE) strains were grown in 96-well plates containing 130 µL liquid GMM at 37°C. Twenty-four replicates were considered per strain. Adherence was measured as described in the Materials and Methods section. The bar graphs represent absorbance readings at 560 nm after (A) 24 h and (B) 48 h. Error bars indicate the standard error. Different letters on the columns indicate values that are statistically different ($p < 0.05$).

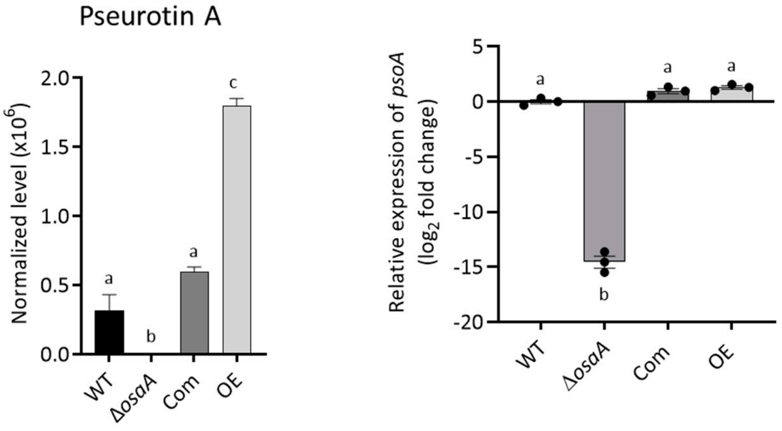

Figure S5. ***osaA* affects production of pseurotin A in *A. fumigatus*.** Wild type (WT), Δ*osaA,* complementation (Com) and overexpression (OE) strains were top-agar inoculated on GMM and incubated at 37°C for 72 h. Extracts were analyzed by liquid chromatography coupled to high resolution mass spectrometry (LC-HRMS). On the right, gene expression of *psoA*, a gene involved in pseurotin A production, was analyzed using the $2^{-\Delta\Delta CT}$ method [68]. WT, Δ*osaA,* Com and OE strains were grown in GMM liquid stationary cultures at 37°C for 48 h. Error bars indicate the standard error. Different letters on the columns indicate values that are statistically different ($p < 0.05$). All the experiments were done in triplicates.

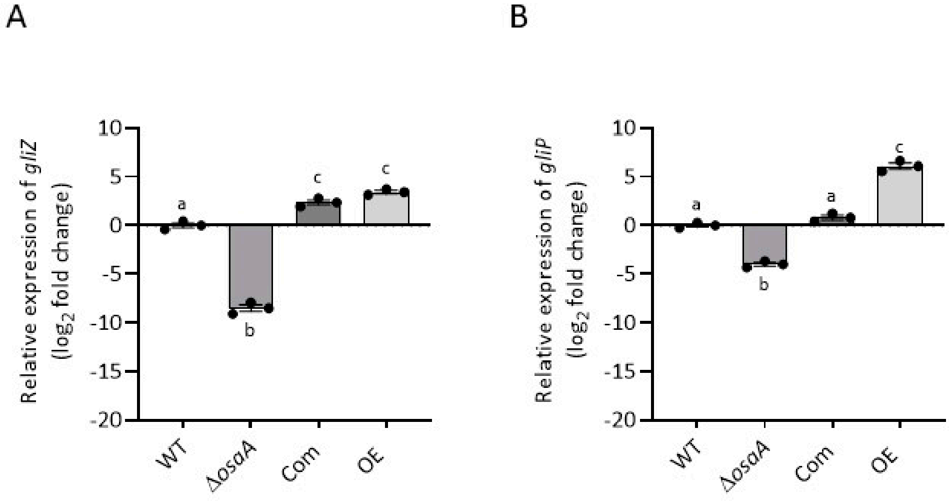

Figure S6. Gene expression of *gliZ* (A) and *gliP* (B) is *osaA*-dependent. Wild type (WT), Δ*osaA,* complementation (Com) and overexpression (OE) strains were grown in liquid shaken YES medium for 72 h at 250 rpm. Expression of genes were analyzed by qRT-PCR and relative expression was calculated following the $2^{-\Delta\Delta CT}$ method [68]. Tubulin gene expression was used as internal reference. Error bars represent standard error. Different letters on the bar represents significantly different values ($p < 0.05$).

Table S1. Primers used in this study.

Name	5'-3' sequence
1. osaA_F1	CCAGGTTGAGACTTCTTTCCCAGG
2. osaA_R2	CCCGAATTGATGGTGGACGTTGT
3. osaA_F3	GCACTCTTCCTACGATTACCCCGT
4. osaa_R4	ACCAAGCAGGATAAAGCTGATCGC
5. osaA_F5	ACAACGTCCACCATCAATTCGGGGATCCTATGGATCTCAG
6. osaA_R6	ACGGGGTAATCGTAGGAAGAGTGCGTCGACATCACCCTTACCC
7. osaA_F7	CACTCCAGCACCAGTTGAACTGTTC
8. osaA_R8	CGGCGTTTTAACTGATGCACCTC
9. A para pyrG_R	CAGGAGCAGCATAAATTCCACGACC
10. osaA_com1	AAGCTTAGTCATCTTCTCGCATGGCGC
11. osaA_com2	GCGGCCGCCATCAAGCGGCTCACATGACCA
12. osaA_OE1	AAAAAAAAGGCGCGCCATGGTGAACGGCACCGCT
13. osaA_OE2	AAAAAAAAGCGGCCGCTTACATTTGCGGCTGAGCTGGAG
14. gpdA _F	AAGTACTTTGCTACATCCATACTCC
15. trpC_R	TGCTTGATCTCGTCTCCCGAAA
16. A fum pyrG_R	GAGCAGCGTAGATGCCTCGAC
17. AfuTub-B_F	TTCCCAACAACATCCAGACC
18. AfuTub-B_R	CGACGGAACATAGCAGTGAA
19. His1	CCGCCGTGGTGGTGTCAAG
20. His2	GGCGTGTTCAGTGTAGGTGACG
21. osaA_CDS_F	CCGTGAGCTTGAAAAGCCGTTCC
22. osaA_CDS_R	TCCACGTCCGACTGATGCGAT
23. fumR_F	CCCAAGACCTCCTCGATCCAGTCCC
24. fumR_R	GGTAGAAAGCCCATCACCAGCATCG
25. pdsA_F	CGCAATACCCTGCACCGACTG
26. pdsA_R	GTTTCGATCTGGAACAGAGCTTCGTTGA
27. Afum_fmqD_F	ACGCTTGGGGGAGGGTTG
28. Afum_fmqD_R	AAGTTCGCACCAGCACCCC
29. gliZ_F_qRT	ACGACGATGAGGAATCGAACCCG
30. gliZ_R_qRT	GGTGCTCCAGAAAAGGGAGTCGTTG
31. gliP_F_qRT	AGTTACACCGACTCGCATCCAGC
32. gliP_R_qRT	CTGGGGCAGACCATGCGTAG
33. Afum_pypC_F	CATCGCACTCGCCTACTCCTTTCA
34. Afum_pypC_R	GCAGTCTTGCTTTGACCGCAT
35. Afum_psoA_F	CTCTGGCGGCGAGATTGGTT
36. Afum_psoA_R	CCGCCCTTCTTTCCATCCTTCC
37. AfumveA_F_new	TGCACCAATATCCGCCAATGC
38. AfumveA_R_new	CGTGTAGGAAGGAGGAGGGGTTACC
39. Afu_brlA_F	GTCAGCAAAGCCGAAGATGGACTACC
40. Afu_brlA_R	GTTGTCGTGGCTCAAGGCGTAC
41. Afum_qPCR_abaA_F	TTGACTCGCTGTCAAGTGATTGTGG
42. Afum_qPCR_abaA_R	TGGTGGATTTGTGGTGGGGAGTT
43. Afu_wetA_F	CTCTACTCACAGCTCTCATCGGGC
44. Afu_wetA_R	GCTGCCATCGTGACTTACTGCG
45. Afu_laeA_F	GTTGCCGTGCGACGAGCAGGA
46. Afu_laeA_R	GGGGCCAAATCAACCCCAACGACG
